# A Conserved Tissue-Specific Homeodomain-Less Isoform of MEIS1 Is Downregulated in Colorectal Cancer

**DOI:** 10.1371/journal.pone.0023665

**Published:** 2011-08-17

**Authors:** Richard C. Crist, Jacquelyn J. Roth, Scott A. Waldman, Arthur M. Buchberg

**Affiliations:** 1 Department of Microbiology and Immunology, Thomas Jefferson University, Philadelphia, Pennsylvania, United States of America; 2 Department of Pharmacology and Experimental Therapeutics, Thomas Jefferson University, Philadelphia, Pennsylvania, United States of America; Virginia Commonwealth University, United States of America

## Abstract

Colorectal cancer is one of the most common cancers in developed nations and is the result of both environmental and genetic factors. Many of the genetic lesions observed in colorectal cancer alter expression of homeobox genes, which encode homeodomain transcription factors. The *MEIS1* homeobox gene is known to be involved in several hematological malignancies and solid tumors and recent evidence suggests that expression of the *MEIS1* transcript is altered in colorectal cancer. Despite this potential connection, little is known about the role of the gene in the intestines. We probed murine gastrointestinal tissue samples with an N-terminal Meis1 antibody, revealing expression of two previously described isoforms, as well as two novel Meis1 products. A 32 kD Meis1 product was expressed in the nuclei of non-epithelial cells in the stomach and colon, while a 27 kD product was expressed in the cytoplasm of epithelial cells in the proximal colon. Our data suggest that the 27 kD and 32 kD Meis1 proteins are both forms of the Meis1d protein, a homeodomain-less isoform whose transcript was previously identified in cDNA screens. Both the *MEIS1D* transcript and protein were expressed in human colon mucosa. Expression of the MEIS1D protein was downregulated in 83% (10/12) of primary colorectal cancer samples compared to matched normal mucosa, indicating that MEIS1D is a biomarker of colorectal tumorigenesis. The decreased expression of MEIS1D in colon tumors also suggests that this conserved homeodomain-less isoform may act as a tumor suppressor in human colorectal cancer.

## Introduction

Colorectal cancer accounts for approximately 10% of both cancer diagnoses and mortalities in the United States (www.cancer.org). The mortality rate has decreased in the previous two decades, primarily reflecting higher levels of compliance with recommended colonoscopy screenings as well as the improved efficacy of therapeutic options [1]; however, colorectal cancer still has the second highest mortality incidence in the United States, trailing only lung cancer. Like many types of cancer, colorectal cancer has both environmental and genetic components [2], [3], [4]. Both sporadic and hereditary forms of the disease are associated with an accumulation of multiple genetic lesions [5], [6], which can result in decreased levels of apoptosis [7], loss of cell cycle regulation [8], and constitutive activation of the *WNT* signaling pathway [9]. As regulators of downstream transcriptional activation, transcription factors are frequently dysregulated in colorectal cancer [10], [11].

Homeobox genes encode proteins with DNA-binding domains known as homeodomains. These homeodomain proteins act as transcription factors, binding promoter regions and activating transcription [12]. *HOX* gene family members are the prototypical homeobox genes. *HOX* genes are involved in embryonic segmentation and patterning as well as the development of numerous organ systems, including the gastrointestinal tract [12]. Developmental genes are often ectopically expressed during carcinogenesis [13] and several *HOX* genes have been linked to colorectal cancer. *HOXB6*, *HOXB8*, *HOXC8*, *HOXC9*, and *HOXD13* are overexpressed in both colorectal cancer cell lines and primary colon tumors [14], [15], [16]. *HOXB13*, however, is normally expressed in colonic mucosa but is downregulated in tumor tissue [10]. Deregulation of non-*HOX* homeobox genes has also been observed in colorectal carcinomas. *CDX1* and *CDX2* are downregulated during colorectal carcinogenesis [17], [18], while aberrant *PROX1* expression in the colon results in increased dysplasia [19].

Homeodomain transcription factors often bind promoter regions as either homodimeric or heterodimeric complexes [20]. This dimerization provides increased specificity of transcriptional activation [21]. The MEIS1 homeodomain protein is a known binding partner of several other homeodomain proteins, including HOXA7, HOXA9, and PBX1 [22]. *MEIS1* plays a role in the normal development of the hematopoietic lineage and is overexpressed in a subset of acute myeloid leukemias [23]. Increased *MEIS1* expression has also been observed in neuroblastomas and the expression level of the *MEIS1* transcript is a prognostic indicator in breast cancer [24]. Downregulation of total *MEIS1* transcript was observed in colorectal adenomas, suggesting a role for *MEIS1* in intestinal tumorigenesis [25].

Due to the links between homeobox genes and colorectal cancer, we examined the status of Meis1 in the colon. In this study, we describe two novel Meis1 products expressed in the murine gastrointestinal tract. These two proteins are expressed in different cell types and subcellular compartments. Both proteins are translated from the *Meis1d* transcript, a homeodomain-less splice variant of *Meis1*. *MEIS1D*, the human homolog of *Meis1d*, was identified in normal human colon tissue at both the mRNA and protein level. Furthermore, we observe the downregulation of *MEIS1D* expression in human colorectal cancers. These data suggest that *MEIS1D* is a novel suppressor of colorectal tumorigenesis and a potential therapeutic target for colon cancer.

## Materials and Methods

### Mouse colony

C57BL/6J (B6) mice were obtained from The Jackson Laboratory (Bar Harbor, ME). Mice were maintained in the AALAC-accredited TJU animal facility. This study was carried out in strict accordance with the recommendations in the Guide for the Care and Use of Laboratory Animals of the National Institutes of Health. The protocol, 343C, was approved by the Institutional Animal Care and Use Committee of Thomas Jefferson University, permit number A3085-01.

### Retroviral Infection

Hct116 cells were obtained from the American Type Culture Collection (Cat. No. CCL-247), where the identity of the cell line was confirmed by STR analysis. MSCV-*Meis1d* and MSCV-*Neo* plasmids were transfected into Phoenix cells using the Profection Calcium Phosphate kit (Promega, Madison, WI). Cells were incubated at 37°C for 24 hours to produce viral media. Viral media was added to Hct116 cells in six well plates and spun at 1,800 rpm for 45 minutes. The cells were then incubated at 32°C for 3 hours, the viral media was removed, and new viral media was added. The cells were spun again at 1,800 rpm for 45 minutes and incubated at 32°C for 3 more hours. Viral media was replaced with DMEM and the cells were moved to 37°C for 48–72 hours to allow translation of the inserted sequence.

### Western blot analysis

B6 mice at 120 days of age were euthanized by CO_2_ asphyxiation followed by cervical dislocation. Tissue and cell samples were washed in 1× PBS and homogenized in lysis buffer (50 mM Tris-HCl, pH 7.4; 150 mM NaCl; 2 mM EDTA; 10% glycerol; 1% Triton-X-100) with protease inhibitors (Roche Applied Science, Indianapolis, IN). Protein concentration was quantified using Bio-Rad Protein Assay (Bio-Rad Laboratories, Hercules, CA). 20 µg of each sample was separated by SDS-PAGE (10% acrylamide) and transferred to nitrocellulose membranes. Membranes were blocked with 5% nonfat dry milk in 1× TBST (20 mM Tris-HCl, pH 7.4; 150 mM NaCl; 0.05% Tween 20). Primary antibodies were used overnight at 4°C: Meis1-N; Meis1d-C; Gapdh (Abcam, Cambridge, MA); actin, histone H1, and cytokeratin 18 (Santa Cruz Biotechnology, Santa Cruz, CA). Horseradish peroxidase-conjugated secondary antibody (Vector Laboratories, Berlingame, CA) was used at a 1∶2500 dilution for 1 hour at room temperature. Proteins were visualized using SuperSignal chemiluminescent substrate (Thermo Scientific, Rockford, IL).

### Subcellular fractionation

Tissue and cell samples were washed in 1× PBS and homogenized in lysis buffer (10 mM Tris-HCl, pH 7.4; 5 mM MgCl_2_; 1 mM DTT; 1 mM PMSF) with protease inhibitors (Roche Applied Science, Indianapolis, IN). Lysates were then passed through a 25½g needle and centrifuged at 600×g at 4°C for 10 min. The supernatant was labeled the cytoplasmic fraction. The pellet was resuspended in lysis buffer and labeled the nuclear fraction.

### Epithelial cell isolation

Proximal and distal colon samples were cut into 1 mm wide strips. The samples were incubated in 1× PBS with 0.15% DTT for 30 min at room temperature with constant agitation by stir bar to remove mucus. Mucosal strips and stir bar were washed in 1× PBS and then incubated in 1× PBS with 1 mM EDTA, pH 7.2 stirring for 60 min at room temperature to remove epithelial cells. The EDTA solution was collected and centrifuged at 470×g for 5 min. Remaining tissue was incubated in the EDTA solution to remove residual epithelial cells and then homogenized in lysis buffer as described above. The epithelial pellet was resuspended in RPMI 1640 and incubated with collagenase (Sigma-Aldrich, St. Louis, MO) at 50 units/mL for 30 min at 37°C, vortexing gently every five minutes. Samples were centrifuged at 200×g for 5 min. The pellet was resuspended in 1× PBS, centrifuged again, and resuspended in RPMI. The RPMI cell suspension was overlaid on a 50% Percoll/PBS mixture. The Percoll gradient was centrifuged at 470×g for 20 min. Epithelial cells were taken from the top of the gradient, diluted with RPMI, and centrifuged at 830×g for 5 min. The cell pellet was resuspended in RPMI and centrifuged again at 470×g for 5 min. The final cell pellet was homogenized in western blot lysis buffer using a 25½g needle.

### RT-PCR

Tissue samples were homogenized in 1 mL TRI Reagent Solution (Ambion, Inc, Austin, TX) and centrifuged at 12,000×g for 15 min at 4°C. 200 µL chloroform were added. The samples were mixed for 15 sec, incubated at room temperature for 3 min, and centrifuged at 12,000×g for 20 min at 4°C. The top layer was mixed with 500 µL of 2-propanol, incubated at room temperature for 10 min, and centrifuged at 12,000×g for 20 min at 4°C. The RNA pellet was washed with 75% ethanol and centrifuged at 7,500×g for 5 min. The RNA was then air dried and resuspended in DEPC-treated water. SuperScript II Reverse Transcriptase (Invitrogen, Carlsbad, CA) was used to generate cDNA. All Meis1 transcripts were amplified in murine samples, while MEIS1D-specific primers were used in human samples.

### Immunohistochemistry

Tissues were fixed in Formalde-Fresh 10% buffered formalin solution (Fisher Scientific) overnight at 4°C and stored in 70% EtOH. Fixed tissues were embedded in paraffin and tissues were cut and mounted on glass slides by the KCC Translational Research Core Facility. Tissues were deparaffinized using citrate buffer, pH 6.0 (Vector Laboratories, Berlingame, CA) and stained with Meis1-N antibody overnight at 4°C. Anti-rabbit secondary antibody (Vector Laboratories, Burlingame, CA) was used at a 1∶200 dilution for 1 hour at 37°C followed by ABC Linker Kit (Vector Laboratories, Berlingame, CA) for 30 min at 37°C. Samples were developed using the DAB Peroxidase Substrate Kit (Vector Laboratories, Burlingame, CA). Staining was visualized on a Nikon Eclipse E600 microscope at either 4× or 20× magnification.

### Ethics

The patients were consented using a written surgical consent form which states the waste from surgery would be used for research purposes. All of the samples were obtained at Thomas Jefferson University Hospital. The Thomas Jefferson University Institutional Review Board (IRB) exempted this study from review because they deemed that the study did not constitute human subjects research and waived the need for consent due the fact that the samples received were coded to remove identification.

## Results

### Two novel Meis1 isoforms are present in the murine gastrointestinal tract

**Figure 1:**
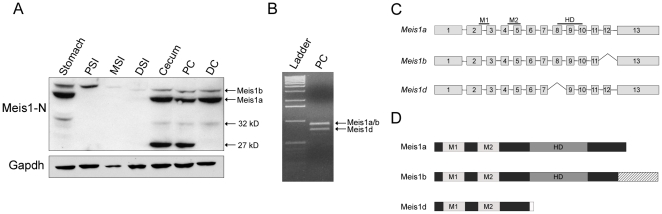
Novel Meis1 isoforms are present in the murine gastrointestinal tract. A) Western blot analysis of B6 gastrointestinal lysates using the Meis1-N antibody. Gapdh was used as a loading control. PSI, MSI, and DSI indicate lysates from the proximal, middle, and distal small intestines, respectively. PC and DC indicate lysates from the proximal and distal colons. B) Amplification of *Meis1* isoforms from B6 proximal colon cDNA using RT-PCR. Diagrams of the C) transcripts and D) products of Meis1a, Meis1b, and Meis1d isoforms are included. The locations of the two Meinox domains (M1 and M2) and the homeodomain (HD) are labeled.

Although downregulation of *MEIS1* in human colorectal cancer has recently been observed, the role of the gene in gastrointestinal (GI) homeostasis and tumorigenesis is poorly understood. To determine the expression of murine Meis1 isoforms in the GI tract, a western blot was performed on C57BL/6J (B6) GI lysates using a polyclonal antibody targeted to the N-terminus of Meis1 (Meis1-N). Products of 43 kD and 51 kD were observed in most samples (Figure 1a). These molecular weights correspond with the known sizes of two previously described isoforms, Meis1a and Meis1b, respectively. Two additional bands were observed in some of the samples: a ∼32 kD protein present in the stomach and colon and a ∼27 kD protein expressed in the proximal colon and cecum (Figure 1a). The ∼27 kD product is the same as the predicted weight for Meis1d, another Meis1 isoform. The transcript for *Meis1d* was previously identified during screens of murine cDNA [26], [27]. The isoform lacks all of exon 8, resulting in a premature stop codon in exon 9 and a theoretical truncated protein product (Figure 1c, d). The existence of an *in vivo* protein product, however, has never been confirmed.

### Meis1d, a truncated Meis1 isoform, is expressed in the murine cecum and proximal colon

If the 27 kD Meis1 product is Meis1d, then the *Meis1d* transcript should be detectable in the proximal colon. To determine if the *Meis1d* transcript is present, *Meis1* splice variants were amplified from B6 proximal colon cDNA samples using RT-PCR. Primers were designed to amplify across exon 8, distinguishing *Meis1d* from the full length isoforms, *Meis1a* and *Meis1b*. The PCR reaction amplified products of approximately 758 and 904 bp in length, the predicted size of products from *Meis1d* and *Meis1a/b* transcripts (Figure 1b). The size difference between the two products corresponds with the known length of exon 8 (146 bp), suggesting that the smaller product is lacking that exon. Extraction and sequencing of the smaller PCR product confirmed the absence of exon 8 from the PCR product (data not shown), demonstrating the presence of the *Meis1d* transcript in the murine proximal colon.

**Figure 2:**
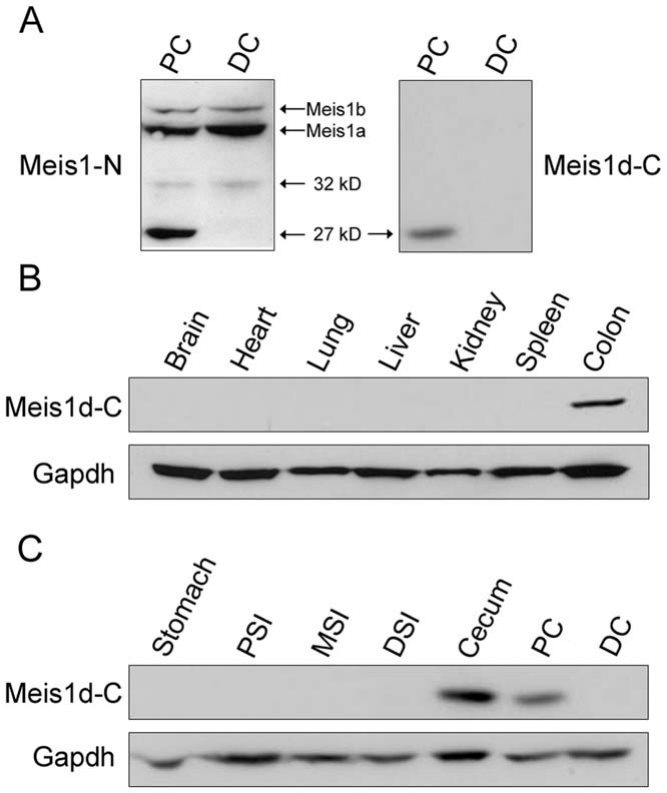
The Meis1d protein is present in the murine colon. A) Western blot analysis of B6 proximal and distal colon lysates with the Meis1-N and Meis1d-C antibodies. The positions of Meis1, Meis1b, and the two novel Meis1 products are labeled. B) and C) Lysates from a panel of B6 organs were probed with the Meis1d-C antibody. Gapdh was used as a loading control.

Removal of exon 8 from the *Meis1d* transcript causes a frameshift in exon 9. The result of this frameshift is the production of truncated protein product with five novel amino acids on the C-terminus (Figure 1d). Since these residues are not present on full length Meis1 isoforms, a custom antibody targeting the unique epitope (Meis1d-C) was generated. To determine if the custom antibody recognizes the ∼27 kD Meis1 product, colon lysates from B6 mice were probed with the Meis1d-C and Meis1-N antibodies. Proximal colon and distal colon lysates were used as positive and negative controls, respectively. As expected, the Meis1-N antibody picked up the previously identified ∼27 kD band in the proximal colon, but not the distal colon (Figure 2a). The same expression pattern was observed in the samples probed with the Meis1d-C antibody, demonstrating that the Meis1d-C antibody detects the ∼27 kD Meis1 product and indicating that the product is Meis1d (Figure 2a). To determine the spatial expression pattern of the Meis1d protein, lysates from a panel of B6 organs were probed with the Meis1d-C antibody. Meis1d expression was limited to the cecum and proximal colon (Figure 2b, c).

### Posttranslational modification of Meis1d correlates with subcellular localization

**Figure 3:**
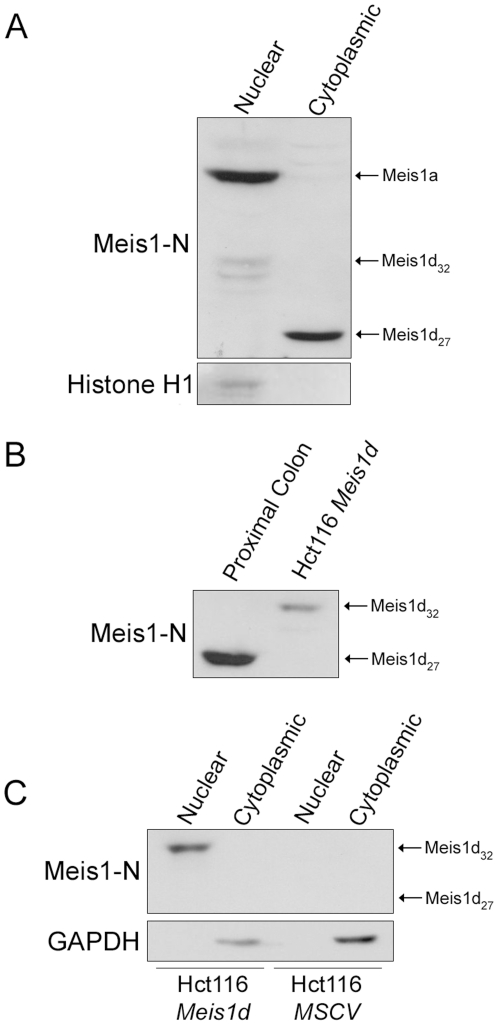
Posttranslational modification of Meis1d correlates with subcellular localization. A) B6 proximal colon samples were separated into nuclear and cytoplasmic fractions and probed with the Meis1-N antibody. Histone H1 was used to confirm the subcellular fractionation. B) Western blot analysis of B6 proximal colon and Hct116 cells expressing the Meis1d ORF using the Meis1-N antibody. C) Hct116 cells were retrovirally infected with the Meis1d ORF or an empty MSCV vector. Cells were taken 72 hours post-infection, fractionated, and probed with the Meis1-N antibody. The positions of Meis1a, nuclear Meis1d (Meis1d_32_), and cytoplasmic Meis1d (Meis1d_27_) are labeled where applicable.

Homeodomain-less isoforms of other homeodomain proteins have been shown to function through two mechanisms. The homeodomain-less proteins can sequester full length isoforms in the cytoplasm through dominant negative interactions. Homeodomain-less isoforms can also act as nuclear binding partners for other transcription factors, altering the activation of downstream target genes. Therefore, the subcellular localization of Meis1d may indicate the functional role of the isoform in the colon. To determine the subcellular localization of Meis1d, B6 proximal colon lysates were separated into nuclear and cytoplasmic fractions and probed with Meis1-N antibody. As expected, full length Meis1 was present in the nuclear fraction (Figure 3a). The 32 kD Meis1 product was also present in the nucleus. The 27 kD Meis1d protein, however, was only observed in the cytoplasmic fraction (Figure 3a). These localization data indicate that Meis1d is not acting as a transcription factor or sequestering full length Meis1 outside the nucleus, suggesting that the isoform has novel cytoplasmic functions.

To study the effects of *Meis1d* expression *in vitro*, the open reading frame (ORF) of *Meis1d* was retrovirally infected into the HCT116 colon cancer cell line. Western blot analysis of cell lysates revealed the presence of a 32 kD protein that was detected by the Meis1-N antibody, but not the Meis1d-C antibody (Figure 3b). This *in vitro* Meis1d product is ∼5 kD larger than the expected 27 kD protein predicted by the Meis1d transcript and observed *in vivo* (Figure 3b). However, the molecular weight matches the novel 32 kD Meis1 isoform previously observed in B6 stomach and colon lysates (Figure 1a). The molecular weight of *in vitro* Meis1d was confirmed in both murine 3T3 and simian Cos-1 cells, indicating that the increased size is not cell line specific (data not shown). Posttranslational modification of the *in vitro* Meis1d product may be the cause of the increased molecular weight, as well as the inability of the Meis1d-C antibody to detect the protein.

Unlike the 27 kD Meis1d product, the 32 kD Meis1 isoform is localized to the nucleus in the proximal colon. To determine if *in vitro* Meis1d is also localized to the nucleus, HCT116 cells expressing Meis1d were fractionated. The 32 kD *in vitro* Meis1d product was present in the nuclear lysates, recapitulating the *in vivo* subcellular localization of the novel 32 kD isoform (Figure 3c). These data suggest that both the 27 kD and 32 kD Meis1 isoforms observed in the murine GI tract are translated from the *Meis1d* transcript. The cytoplasmic form (Meis1d_27_) is the predicted molecular weight of Meis1d, while the nuclear form (Meis1d_32_) is larger, most likely due to posttranslational modification.

### Nuclear and cytoplasmic Meis1d are mutually exclusive in the colon

**Figure 4:**
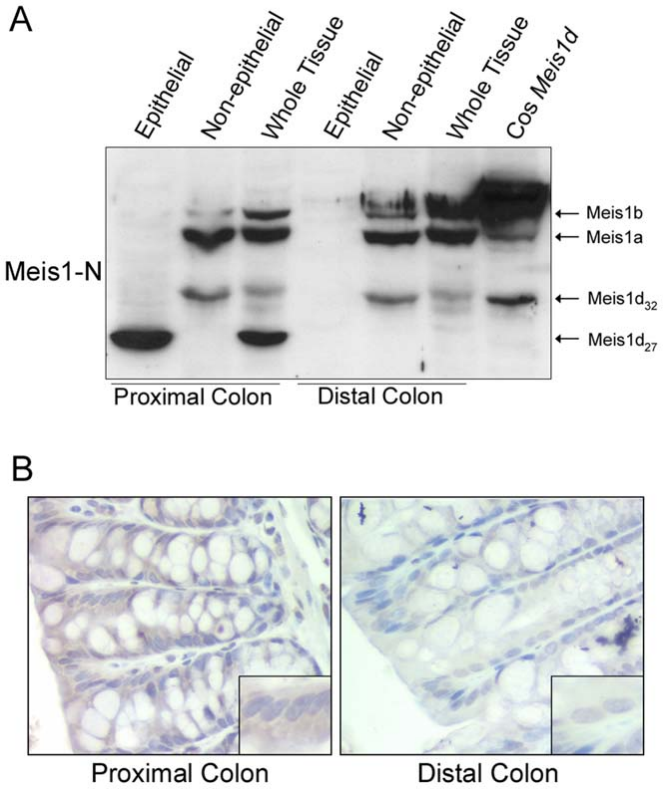
Meis1d_27_ and Meis1d_32_ are expressed in different cellular populations. A) B6 proximal and distal colon samples were separated into epithelial and non-epithelial fractions. The non-epithelial fractions consist of the lamina propria and muscle layers of the colon. The fractions and whole cell lysates from both colon segments were probed with the Meis1-N antibody. Cos-1 cells expressing Meis1d were used to compare the *in vitro* and *in vivo* molecular weights of Meis1d_32_. B) B6 proximal and distal colon samples were stained with Meis1-N antibody. Staining was visualized at 20× magnification.

The colon consists of a single layer of epithelial cells covering the lamina propria and several muscle layers. Colon cancer is derived from stem cells located in the epithelial layer [28]. To determine if either Meis1d_27_ or Meis1d_32_ are expressed in the colonic epithelium, epithelial cells were isolated from B6 proximal and distal colon samples. Lysates from the isolated epithelial cells and lamina propria/muscle layers were probed with Meis1-N antibody. The Meis1d_27_ protein was expressed exclusively in the epithelial cells of the proximal colon (Figure 4a). Meis1d_32_, however, was expressed in the lamina propria/muscle layers from both the proximal and distal colon (Figure 4a). The full length Meis1 isoforms, Meis1a and Meis1b, were also present in only the lamina propria/muscle samples (Figure 4a). These data indicate that the nuclear and cytoplasmic forms of Meis1d are not present in the same cell populations in the murine colon. Furthermore, Meis1d_27_ is expressed in colonic epithelial cells in the absence of full length Meis1. To further confirm the epithelial cell expression of Meis1d_27_, B6 colon samples were stained with Meis1-N antibody. Strong cytoplasmic staining was observed in the epithelium of the proximal colon, but not the distal colon (Figure 4b). This expression pattern recapitulates the expression of Meis1d_27_, again suggesting that Meis1d_27_ is present in the epithelial cells of the proximal colon.

### Expression of the MEIS1D protein is downregulated in human colorectal cancers

**Figure 5:**
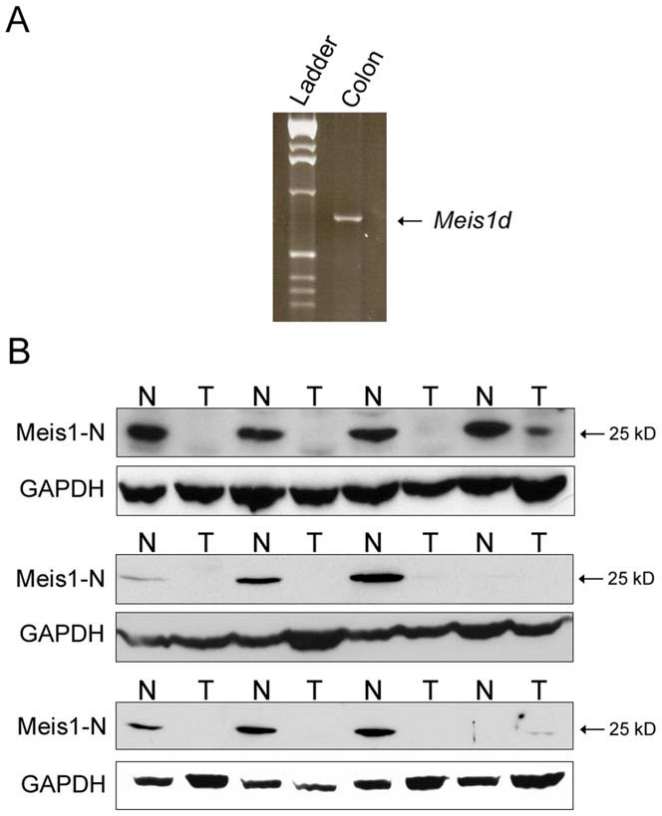
MEIS1D_27_ is downregulated in human colorectal cancers. A) RT-PCR amplification of *MEIS1D* transcript from human colon cDNA. B) Western blot analysis of lysates from human colorectal tumors (T) and matched normal mucosa (N) using the Meis1-N antibody. GAPDH was used as a loading control. Samples represent all four sections of the human colon: ascending, transverse, descending, and sigmoid.

Meis1 is highly conserved in eukaryotes and removal of exon 8 from the human MEIS1 mRNA would encode a predicted protein product with 99% homology to murine Meis1d. Based on the evolutionary conservation of Meis1, we attempted to characterize the expression of MEIS1D in the human colon. A western blot was performed on human colon lysates using the Meis1-N antibody. A band the predicted size of MEIS1D_27_ was observed in human colon samples (Figure 5b). To confirm the expression of the MEIS1D transcript, RT-PCR was performed on human colon cDNA (Figure 5a). A 758 bp band was amplified, indicating that *MEIS1D* mRNA is transcribed in the human colon.

Expression of full length MEIS1 is known to be dysregulated in several solid tumors [29], [30]. To determine the status of MEIS1 in colorectal cancer, western blots were performed on matched normal colon and primary colorectal tumor lysates using the Meis1-N antibody. MEIS1A and MEIS1B were expressed in both tumor and normal samples, while MEIS1D_32_ was not present in either set of samples (data not shown). However, 83% (10/12) of normal colon mucosa samples expressed detectable levels of MEIS1D_27_ protein (Figure 5b). Expression was reduced or completely lost in all matched tumor samples from these patients (Figure 5b). The remaining two patients expressed undetectable levels of MEIS1D_27_ in both normal mucosa and tumor tissue (Figure 5b). These *in vivo* data indicate that MEIS1D_27_ expression is downregulated during intestinal tumorigenesis.

## Discussion

Alternative splicing is known to produce two *Meis1* isoforms, *Meis1a* and *Meis1b*, in both mice and humans. Both the Meis1a and Meis1b proteins are full length, containing the two Meinox domains involved in protein-protein interactions and the DNA-binding homeodomain (Figure 2d). The protein products of these splice variants differ in the C-terminus, due to the splicing out of exon 12 from the *Meis1b* coding sequencing (Figure 2c, d). Evidence suggests that the two similar isoforms may activate transcription of different subsets of genes [31]. Screens of cDNA libraries indicate that a large number of additional alternatively spliced transcripts are produced from the *Meis1* locus [26], [27], [32]. Unlike *Meis1a* and *Meis1b*, however, proteins for other *Meis1* transcripts have not been observed *in vivo*. Therefore, it remains unclear whether these transcripts are functionally relevant or simply artifacts generated by incorrect alternative splicing.

In this paper, we have described two protein products of the *Meis1d* splice variant, an isoform previously identified in cDNA screens. The *Meis1d* transcript lacks exon 8, resulting in a predicted 27 kD protein lacking the homeodomain (Figure 2c, d). This 27 kD product was observed in the cytoplasm of proximal colon epithelial cells (Figures 3a, 4a). A second, 32 kD Meis1d product occurs in the nuclei of non-epithelial cells in the stomach and colon (Figures 3a, 4a). The cytoplasmic and nuclear forms of Meis1d have been named Meis1d_27_ and Meis1d_32_, respectively. The larger molecular weight and nuclear localization of the Meis1d_32_ protein are recapitulated in cells transfected with the *Meis1d* ORF (Figure 3b). Expression of MEIS1D_27_ in human colon samples was also observed, demonstrating evolutionary conservation of this novel isoform (Figure 5). *Meis1d* is only the third *Meis1* isoform for which a translated protein product has been confirmed and is also the first homeodomain-less Meis1 product observed in either mice or human tissues.

Homeodomain-less isoforms have been described for many homeobox genes, including several members of the Hox family. Only a few of these isoforms, however, have been identified in TALE superfamily genes, a group including Meis1. The loss of the homeodomain prevents direct binding to DNA target sequences and normal transcriptional activation. Homeodomain-less isoforms of TALE transcription factors, however, have been previously shown to still regulate transcription through two distinct mechanisms. The truncated isoforms can have dominant negative effects, sequestering full length proteins in the cytoplasm and preventing transcriptional activation of downstream targets [33], [34]. The homeodomain-less proteins can also interact indirectly with DNA by binding other transcription factors to form heterodimers. The presence of the truncated proteins alters the DNA binding site of the protein complex, activating a distinct subset of downstream targets [35].

Since homeodomain-less isoforms of related genes have distinct mechanisms in the cytoplasm and nucleus, the subcellular localizations of Meis1d and any potential binding partners may suggest a cellular function. In the present study, Meis1d was observed in either the nucleus or cytoplasm depending on cell type. Meis1d_27_ is expressed in the cytoplasm of proximal colon epithelial cells (Figures 3, 4). Full length isoforms are present in the nuclear fraction of proximal colon lysates, indicating that Meis1d_27_ does not sequester other Meis1 isoforms in the cytoplasm (Figures 3, 4). Further evidence indicates that Meis1d_27_ is not expressed in the same cells as Meis1a or Meis1b, preventing any kind of dominant negative interaction (Figure 4). The cytoplasmic form of Meis1d may still bind other unknown transcription factors and prevent nuclear localization. The isoform may also have a novel homeodomain-independent function unrelated to transcriptional activation.

The potential function of Meis1d_27_ is unclear because the data does not fit either known mechanism of homeodomain-less TALE proteins. The protein can not be acting as a dominant negative, since it is not expressed in the same cells as other Meis1 isoforms. Meis1d_27_ also can not be activating downstream transcription inside the nucleus, because the protein is localized to the cytoplasm. The nuclear form of Meis1d, however, may still fit either of these mechanisms in the lamina propria or muscle surrounding the colon. The nuclear localization of Meis1d_32_ means the protein could be acting as a binding partner for other transcription factors. Meis1d_32_ still maintains the motifs required for Pbx interaction and homodimerization [36]. These data suggest that the truncated isoform could still function as a co-factor with these other homeodomain proteins. The Meis1d_32_ protein may also be acting as dominant negative isoform inside the nucleus, preventing association between full length Meis1 proteins and promoter regions in the DNA. Further dissection of the lamina propria and the underlying muscle layer is necessary to determine if Meis1d_32_ and any potential binding partners are expressed in the same population of cells. Identification of functional roles for both Meis1d_32_ and Meis1d_27_ will help explain the relevance of *Meis1* splicing in intestinal function and homeostasis.

Despite the lack of a defined function, we have provided evidence that MEIS1D_27_ is downregulated in primary colorectal cancers compared to normal mucosa (Figure 5b). These data may explain the downregulation of total MEIS1 transcript previously observed in early colon adenomas [25]. Preliminary data also suggests that MEIS1D_27_ is not expressed in the Hct116, LoVo, or SW480 cell lines, which are derived from human colon cancers (data not shown). Tumor suppressor genes are functionally inactivated during tumor initiation and progression through somatic mutations or loss of expression. These data suggest that MEIS1D_27_ may act as a tumor suppressor in the colon. The loss of MEIS1D_27_ during intestinal tumorigenesis, however, may also be indicative of more global changes in the transcriptome of the tumor cell. Alternative splicing is an essential mechanism for generating the protein diversity necessary for cellular function. Splicing provides both direct and indirect regulation of multiple cancer-related pathways, including apoptosis, signal transduction, and differentiation [37], [38], [39]. As with other regulatory mechanisms, alterations in alternative splicing have been observed in primary human cancers [40], [41]. Transcriptome-wide changes are often caused by aberrant expression of splicing factors, resulting in simultaneous downregulation of tumor suppressive isoforms and upregulation of oncogenic isoforms. Newer microarrays can map the transcriptome with exon-level resolution, allowing analysis of these changes in primary tumors. Despite these technological advancements, there are still difficulties identifying causative links between production of specific isoforms and tumorigenesis. Due to the global changes in alternative splicing observed in cancer, MEIS1D may be one of many splice variants whose expression levels change during intestinal tumorigenesis. Future work will need to determine if there is a causative role for MEIS1D_27_ in colorectal cancer or if the loss of the cytoplasmic isoform is simply a biomarker of colorectal cancer.
